# Uses of Electricity

**Published:** 1893-03

**Authors:** 


USES OF ELECTRICITY.—America has become the great centre of the production of electric light and power. A backward glance over a few years shows how young all the work is, The advantage of incandescent lighting need hardly be enlarged upon to-day. The field of electric traction is great; the field of electric mining is even more extensive. The extent to which the idea of electric power in factories has won the day is shown in the numerous shops now operating in this way. In trolley cars the electric heater is becoming well nigh as common an object as incandescant lamps. The application of electricity in dental practice, adapted to the dental engine, the electric mallet, the mouth lamp, is well known to readers of this journal.

But " electricity up to date " would not be complete without reference to modern electro-therapeutics. It was the privilege of the writer recently to enjoy an " electric steam bath," given by Dr. Geo. W. Barkman, Hamilton Terrace. Baltimore. Dr. Barkman's electric treatment has been practically tested by gentlemen well and favorably known in Baltimore. The benefits to be gained from the use of electricity when applied by experienced hands in properly selected cases, and in nearly all forms of nervous diseases, are just becoming known. The cases that may be treated (but not by electricity exclusively) cover a wide range, including all kinds of neuralgias, facial and intercostal; gastralgia, cardialgia, sciatica, lumbago, etc.; hemicrania and all varieties of headache; the various paralyses in all possible situations; general nervous affections, both functional and inorganic, such as hysteria, hypochondria, paralysis agitans, progressive muscular atrophy, locomotor ataxia. In this connection it is well to emphasize its use in cases of facial neuralgia which come under the notice of dentists, as its application has met with gratifying results.

Dr. Hammond is quoted as saying: " Many of our physicians know almost nothing of this great agent." Would it not, therefore, it may be suggested in conclusion, be becoming in the family physician who has never been taught its use, who has no proper apparatus and would not know how to use it if he had, to place suitable cases in skillful hands? There can be no doubt that Dr. Barkman understands the working principles of the batteries he uses; in fact, he is an expert electrician able to construct and repair them. R. G.

